# On the intrinsic disorder status of the major players in programmed cell death pathways

**DOI:** 10.12688/f1000research.2-190.v1

**Published:** 2013-09-17

**Authors:** Alexey V Uversky, Bin Xue, Zhenling Peng, Lukasz Kurgan, Vladimir N Uversky

**Affiliations:** 1 Center for Data Analytics and Biomedical Informatics, Department of Computer and Information Sciences, College of Science and Technology, Temple University, Philadelphia, PA, 19122, USA; 2 Department of Molecular Medicine, College of Medicine, University of South Florida, Tampa, FL, 33612, USA; 3 Department of Electrical and Computer Engineering, University of Alberta, Edmonton, Canada; 4 Byrd Alzheimer's Research Institute, College of Medicine, University of South Florida, Tampa, FL, 33612, USA; 5 Institute for Biological Instrumentation, Russian Academy of Sciences, 142290 Pushchino, Russian Federation

## Abstract

Earlier computational and bioinformatics analysis of several large protein datasets across 28 species showed that proteins involved in regulation and execution of programmed cell death (PCD) possess substantial amounts of intrinsic disorder. Based on the comprehensive analysis of these datasets by a wide array of modern bioinformatics tools it was concluded that disordered regions of PCD-related proteins are involved in a multitude of biological functions and interactions with various partners, possess numerous posttranslational modification sites, and have specific evolutionary patterns (Peng *et al*. 2013). This study extends our previous work by providing information on the intrinsic disorder status of some of the major players of the three major PCD pathways: apoptosis, autophagy, and necroptosis. We also present a detailed description of the disorder status and interactomes of selected proteins that are involved in the p53-mediated apoptotic signaling pathways.

## Introduction

Many biologically active proteins do not have a unique 3-D structure as a whole or in part ^1– 5^, and are as such described as intrinsically disordered proteins (IDPs) and hybrid proteins containing ordered domains and IDP regions (IDPRs). These proteins and regions possess highly flexible structures and exist as conformational dynamic ensembles characterized by different degree and depth of disorderedness ^2, 4, 6– 10^. Globally, the structure of IDPs/IDPRs can be described as collapsed-disordered (molten globule-like), partially collapsed-disordered (pre-molten globule-like), or extended-disordered (coil-like) ^8, 11^. IDPs/IDPRs are highly abundant in virtually any given proteome ^1, 3, 5, 12^. As they are abundant constituents of all cells, tissues, and organs, and are responsible for crucial controling and regulating functions, IDPs are commonly involved in the pathogenesis of various human diseases ^13^. This conclusion is based on numerous case studies in which a particular IDP was shown to be associated with a particular disease (including many cancer- ^14– 19^ and neurodegeneration-related proteins ^20– 27^), as well as on the results of systematic bioinformatics studies ^12, 28– 36^.

The phenomenon of intrinsic disorder in proteins is spreading through modern protein science, and is implemented in more and more aspects of protein functionality. The biological functions of IDPs/IDPRs include envolvement in regulation, signaling, and controlling pathways ^29, 37, 38^. These disorder-based functions represent a crucial complementation to the functional repertoire of ordered proteins ^30, 39– 41^. Conformational plasticity, pliability, and adjustability combined with functional versatility define the overall natural abundance of IDP/IDPRs. As a result, many (if not the vast majority) of the non-catalytic functions of proteins frequently rely on the advantages provided by the lack of a fixed unique structure ^30, 39– 41^.

In addition to being crucial for the functionality of many individual proteins, intrinsic disorder plays a vital role in the control of almost all cellular processes. For example, in our recent study, we analyzed the abundance and roles of intrinsic disorder in proteins involved in the various pathways related to programmed cell death (PCD) ^42^. PCD represents a suicidal cellular response to the exposure to a set of environmental factors that trigger a chain of specific intracellular biochemical events leading to characteristic morphological cellular changes, and ultimately to cell death.

The three PCD routes (or PCD types I, II and III, which correspond to apoptosis, autophagy, and necroptosis, respectively) have very different biological roles. Apoptosis is important for the development, immune regulation, and homeostasis of a multi-cellular organism. Necroptosis plays a role in the modulation of the inflammatory response in the skin and intestine, serves as a backup mechanism to clear pathogens, and is involved in the immunologically silent maintenance of the T cell homeostasis ^43^. Autophagy controls a wide range of physiological processes such as starvation, cell differentiation, cell survival, and death ^44– 46^. It regulates the turnover of long-lived proteins, the disposal of damaged organelles and misfolded proteins, and the turnover of cellular building blocks following nutrient deprivation. As a result, autophagy typically has crucial pro-survival roles in cellular homeostasis and during stress. However, under some circumstances, it can initiate characteristic cell death ^44, 47^. Therefore, although apoptosis and necroptosis both invariably contribute to cell death, autophagy might play either pro-survival or pro-death roles ^47– 49^. As a result, the fate of the cells and the fine balance between cell death and survival of healthy cells is jointly decided by the interplay between these three PCD pathways ^49^.

Since these three major PCD mechanisms involve different signaling pathways, they can be easily distinguished from each other based on their specific morphological features and on the unique biochemical changes developing in dying cells ^47, 50^. Among the characteristic morphological features of the three PCD types are the apoptosis-specific fragmentation and condensation of chromatin, combined with the fragmentation of chromosomal DNA, characteristic fragmentation of nucleous, shrinkage of cells, and production of cell fragments known as apoptotyc bodies ^49^; the autophagy-linked formation of autophagosomes, which are the double- or multimembrane-bound structures around the recycling-destined cytoplasmic macromolecules and organelles ^49, 51– 55^; and the necroptosis-related dysfunction of organnelles accompanied by the swelling and lysis of cells ^56^.

Although a wide range of cell signals of either extracellular or intracellular origin can lead to the activation of various pathways eventually resulting in the initiation of apoptosis, the major cause of the cell death is related to the organized degradation of cellular organelles by activated members of the caspase family of cysteine proteases ^57^. Depending on the origin of the triggering signal, apoptotic pathways are classified as extrinsic and intrinsic, and each of these pathways can be regulated at multiple levels. Furthermore, depending on the nature of the cell signals, both apoptotic pathways can be either initiated or repressed. Among the extracellular (or extrinsic) inducers of apoptosis are various cytokines, nitric oxide ^58– 60^, hormones, growth factors, and toxins ^61^, which somehow cross the plasma membrane or transduce to affect a response. Among the major players of the extrinsic apoptosis pathway are cell surface receptors known as death receptors, decoy receptors serving as inhibitory counterparts of these death receptors, are a set of related cytoplasmic proteins ^62^. Furthermore, this pathway is regulated by changes in the transcription levels of the death inhibitory proteins (e.g., FLIP) and by the variations in the expression levels of the specific cytoplasmic adapter proteins, such as FADD and other apoptosis-activating ligands, leading to the procaspase activation ^62, 63^. Among the intracellular triggers of apoptosis are specific signals released by a damaged cell in response to different types of stress, such as heat, radiation, nutrient deprivation, increased intracellular calcium concentration ^64^, hypoxia, or viral infection. The intrinsic pathway centers on the mitochondria that contains several key apoptogenic factors such as cytochrome *c*, AIF, SMAC/DIABLO, Htra2/Omi ^65, 66^, and endoG ^67– 69^; the release of these factors from mitochondria is regulated by the pro- and anti-death members of the BCL-2 family ^70^. Also, members of the inhibitor of apoptosis protein (IAP) family of functionally and structurally related proteins control both intrinsic and extrinsic apoptosis pathways, serving as endogeneous apoptosis inhibitors ^62^. Finally, a unique regulatory role is played by a transcription factor p53 via its modulation of many key control points in both the extrinsic and intrinsic pathways ^62^.

Among the major molecular players of autophagy are the mammalian target of rapamycin kinase (mTOR), the ULK1 kinase complex ^32^, the class III PI(3)-kinase VPS34, Beclin-1 ^31^, and several members of the ATG family that possess various biological functions ^47^. Regulators of necroptosis are specific to cell types being different for the apoptosis-competent and the apoptosis-incompetent cells. In fact, RIP1 kinase and death receptors play a role in activation and control of the necroptosis in the apoptosis-incompetent cells ^71^, whereas the necroptosis of the apoptosis-competent cells is regulated by reactive oxygen species, apoptosis inducing factor (AIF), death-associated protein kinase (DAPK), and c-Jun N-terminal kinase (JNK) ^72– 75^.

Earlier we reported the results of a comprehensive computational analysis of 1138 human apoptosis-related proteins, 137 human proteins associated with and autophagy, and 35 human necroptosis-related proteins, and also studied 3,458 proteins from DeathBase ^76^ ( http://deathbase.org/) that included proteins from five manually curated species: human, mouse, zebrafish, *D. melanogaster*, and *C. elegans*, and 23 reference species ^42^. This previous analysis revealed that proteins involved in the regulation and execution of PCD possess substantial amounts of intrinsic disorder; that disorder has numerous functional roles across and within apoptosis, autophagy, and necroptosis processes; and that IDPRs of the PCD-related proteins are engaged in protein-protein interactions, interactions with other partners including nucleic acids and other ligands, and are also enriched with posttranslational modification sites ^42^. In the current study, we extend our previous work and provide information on the intrinsic disorder status of some of the major players of the three major PCD pathways: apoptosis, autophagy, and necroptosis. The extention is related to the more focused consideration of the disorder status of several specific human proteins related to the PCD pathways and consideration of the roles of disorder in functions of these proteins;, aspects not covered in the previous article. We also present a detailed description of the disorder status and interactomes of selected proteins that are involved in the p53-mediated apoptotic signaling pathways, which was not covered in the previous article. Finally, we provide analysis of available structural information for human proteins related to the p53-controlled apoptotic pathways.

## Methods

### Datasets

**Figure 1.**
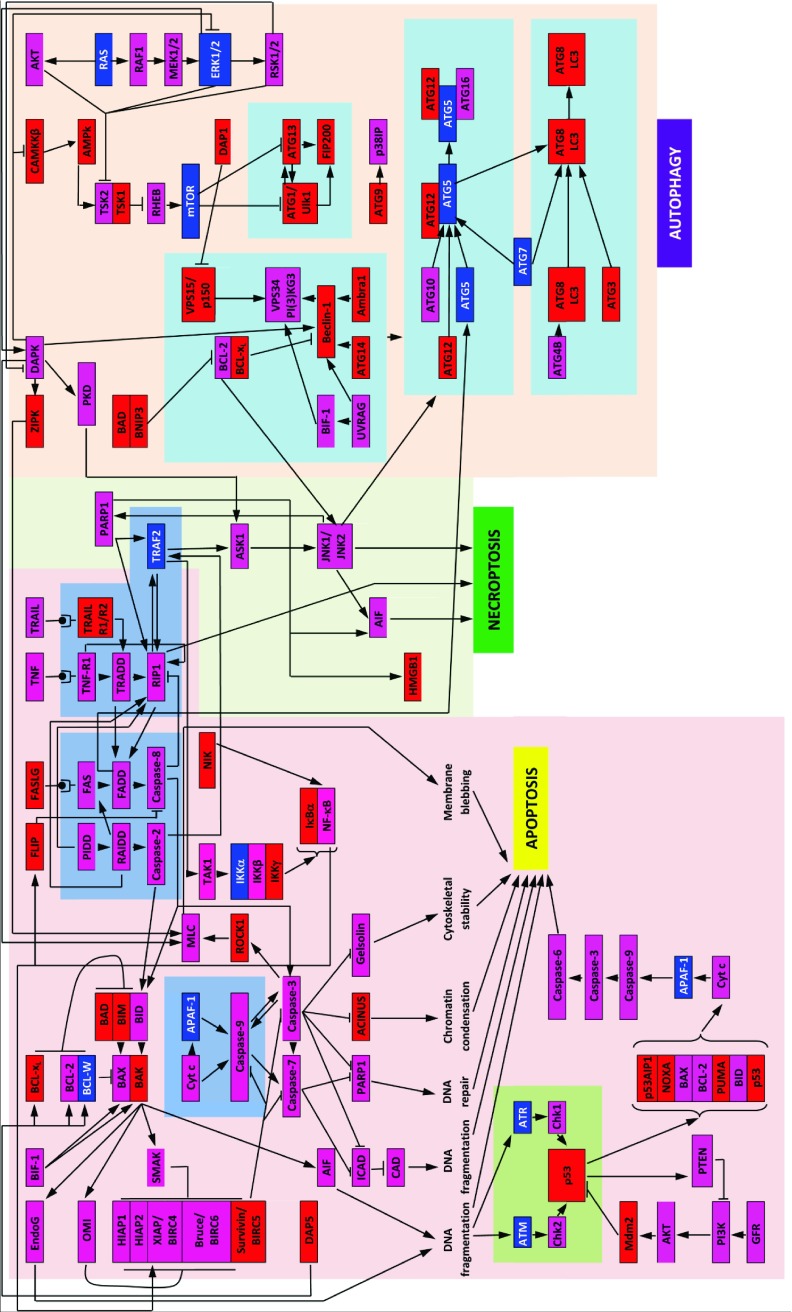
Schematic representation of three PCD modules. Diagram shows a map of the regulators and molecular components of the apoptosis, autophagy, and necroptosis death pathways, constituting the three known modules of the programmed cell death (PCD) network. The proteins are color coded according to their intrinsic disorder content evaluated by PONDR-FIT, with highly ordered ([IDP score]<10%), moderately disordered (10%≤ [IDP score]<30%), and highly disordered ([IDP score]≥30%) being shown as blue, pink, and red boxes, respectively. This diagram is based on the PCD maps published in ^47, 150, 151^ and is reproduced from ^42^.

**Figure 2.**
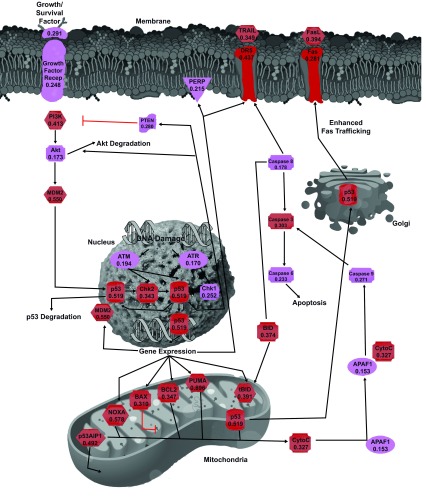
Schematic representation of the p53-mediated apoptotic signaling pathways. Diagram shows a map of the regulators and molecular components of this pathway. The proteins are color coded according to their intrinsic disorder content evaluated by PONDR-FIT, with highly ordered ([IDP score]<10%), moderately disordered (10%≤ [IDP score]<30%), and highly disordered ([IDP score]≥30%) being shown as blue, pink, and red boxes, respectively. This diagram is based on the maps of the p53-mediated apoptotic signaling pathways available at ( http://www.ebioscience.com/resources/pathways/p53-mediated-apoptosis-pathway.htm) and ( http://www.qiagen.com/Products/Genes and Pathways/Pathway Details/?pwid=338) and on related information ^62, 109, 151, 152^ and many other papers.

In this study we extend the previous analysis of the abundance and roles of intrinsic disorder in the control and execution of PCD pathways ^42^. We report the results of the focused evaluation of intrinsic disorder in specific sub-sets of human proteins in three major modules of the programmed cell death, namely apoptosis, necroptosis, and autophagy. The analyzed proteins are shown in Figure 1 which represents an interlinked network of protein-protein interactions related to PCD. We also paid special attention to 29 human proteins related to the p53-mediated apoptotic pathways (see Figure 2). All human proteins considered in this study were reviewed entries in the UniProtKB Protein Knowledgebase ( http://www.uniprot.org/uniprot/), and the corresponding FASTA files were downloaded from UniProt.

### Computational characterization of disorder

The intrinsic disorder status in these proteins was evaluated by PONDR FIT ( http://www.disprot.org/pondr-fit.php) ^77^, which is a meta-predictor that combines six individual predictors: PONDR ^®^ VLXT ^78^, PONDR ^®^ VSL2 ^79^, PONDR ^®^ VL3 ^80^, FoldIndex ^81^, IUPred ^82^, TopIDP ^83^ and by PONDR ^®^ VLXT ^78^. PONDR FIT is somewhat more accurate than the individual predictors used for its development ^77^. We also analyzed the disorder status of human protein related to the p53-mediated apoptosis signaling pathways by PONDR ^®^ VLXT. The use of PONDR ^®^ VLXT is determined by the ability of this computational tool to visualize potential functional sites important for molecular recognition, signaling, and regulation. These potential binding sites, which are now recognized as Molecular Recognition Features (MoRFs) ^84, 85^, occur as dips on the plot of disorder score, and correspond to segments with an increased propensity towards order that are flanked by disordered regions.

### Finding α-helix-forming molecular recognition features (α-MoRFs)

MoRFs are short order-prone motifs flanked by disordered regions that are involved in molecular recognition and are able to undergo disorder-to-order transition during binding to a specific partner. The recognition capability of MoRFs and their ability to undergo induced folding at binding are defined by their specific amino acid biases, since these regions usually have a much higher content of aliphatic and aromatic amino acids than disordered regions in general and therefore are computationally identifiable ^84, 85^. We used α-MoRF-Pred and α-MoRF-Pred II tools to find α-helix-forming MoRFs ^84, 85^.

### ANCHOR analysis

The ANCHOR algorithm represents an alternative approach for finding potential binding sites in IDPs and IDPRs ^86, 87^ ( http://anchor.enzim.hu/). At the foundation of this approach and its parental tool, the general disorder predictor IUPred ^82, 88^, is the estimation of the pairwise energy. Here, the potential binding sites are found as protein segments that cannot form enough favorable intra-chain interactions to fold on their own, but are likely to gain stabilizing energy via specific interactions with globular protein partners ^86, 87^. In line with our earlier studies ^89– 91^, these potential binding regions are termed here as ANCHOR-indicated binding site (AIBS).

### STRING analysis

Finally, we looked at the interactions of the functional analysis of these proteins using the STRING database ^92^. The STRING database ( http://string-db.org), which covers more than 1100 completely sequenced organisms, including *Homo sapiens*, represents the online database resource search tool for the retrieval of interacting genes providing both experimental and predicted interaction information ^92^. For each protein, STRING produces the network of predicted associations for a particular group of proteins. The network nodes are proteins. The edges represent the functional associations evaluated based on experiments, search of databases, and text mining. The thickness of edges is proportional to the confidence level ^92^. We used two sets of parameters in this analysis. To show the breadth of the p53-centered interactome, the number of interactors was set to 500 and the STRING confidence level was set to 0.900. In the analysis of other human proteins involved in the p53-controlled apoptotic signaling pathways, the number of interactors was set to 20 and the medium confidence level of 0.4 was used.

## Results and discussion

Figure 1 (which is adopted from Peng *et al.* ^42^) schematically represents the interlinked nature of the three PCD-related modules by showing some major events taking place within a cell undergoing apoptosis, autophagy, or necroptosis ^47^. Figure 1 clearly shows that the various programmed cell death processes are under tight control and that the involved proteins are strongly interconnected. In fact, it is clear that inside the challenged cell, there is a common programmed cell death network that integrates three PCD modules, which include many pathways that are intertwined and interconnected, and many death regulatory proteins that are used by more than one module ^47^. Our previous study suggested that one of the common structural features of these PCD-related proteins is their strong propensity for being disordered or to possess long IDPRs ^42^. We believe that this disordered nature of PCD-controlling proteins allows them to be uniquely and effectively modulated via multiple specific interaction with various partners effectively, and to control the regulation and execution of different PCD modules ^42^.

This conclusion is supported by a simple visualization technique used in Figure 1, namely, coding the involved proteins according to their intrinsic disorder content evaluated by PONDR-FIT. We used two arbitrary cutoffs for the levels of intrinsic disorder to classify proteins as highly ordered ([IDP score]<0.1, blue boxes), moderately disordered (0.1≤ [IDP score]<0.3, pink boxes) and highly disordered ([IDP score]≥0.3, red boxes) ^36^. According to this classification, only 11 human proteins related to the controlled cell death pathways shown in Figure 1 are characterized by low disorder scores, whereas the absolute majority of the PCD-related proteins are moderately or highly disordered. Figure 1 also shows that the highly connected PCD-related proteins (i.e., proteins involved in several functional interactions) are typically more disordered than proteins with a lesser number of interaction partners. Interestingly, Figure 1 also illustrates that although many of the human PCD-related proteins are enzymes (kinases, ribonucleases, deoxyribonuclease, proteases, protein and ubiquitin ligases, polymerases, oxidureductase, GTPases, etc.), they possess significant disorder levels. The discussion below provides a very brief overview of some major players in three PCD modules and their disorder status.

### Description of some major players in three PCD modules and their disorder status

The paragraphs below provide a more focused description of some major functions ascribed to the proteins in the three PCD modules shown in Figure 1. This description is further enhanced by our analysis of the disorder status of these proteins, where all mean PONDR-FIT scores were determined in this study.

Apoptosis is a type I programmed cell death that is essential for the elimination of unwanted cells during normal development, and for the maintenance of tissue homeostasis. Apoptosis starts with the formation of multiprotein complexes. One of these complexes is the extrinsic pathway-specific death-inducing signaling complex (DISC) triggered by binding of extracellular death ligands to death receptors. Typical DISC consists of the death receptor FAS (mean PONDR FIT score of 0.281), death ligand (mean PONDR FIT score of 0.394), adaptor proteins such as FADD (mean PONDR FIT score of 0.274) and TRADD (mean PONDR FIT score of 0.298) ^93^. Another important complex formed at these early apoptosis stages is the intrinsic pathway-specific apoptosome, consisting of APAF1 (mean PONDR FIT score of 0.154) and cytochrome *c* (mean PONDR FIT score of 0.327), formation of which is triggered by the cytochrome *c* release from the mitochondria ^94, 95^. At the next stage, initiator caspases (such as caspase-2 (mean PONDR FIT score of 0.122), caspase-8 (mean PONDR FIT score of 0.178), and caspase-9 (mean PONDR FIT score of 0.271)) are recruited and activated by these initial complexes. These secondary complexes then cleave and activate effector caspases, including caspase-3 (mean PONDR FIT score of 0.303) and caspase-7 (mean PONDR FIT score of 0.221), that target specific cellular substrates for proteolysis ^57^. Caspases are directly inhibited by members of the IAP family (such as HIAP1 (mean PONDR FIT score of 0.212), HIAP2 (mean PONDR FIT score of 0.215), XIAP (mean PONDR FIT score of 0.177)), as well as by Bruce (mean PONDR FIT score of 0.223) and Survivin (mean PONDR FIT score of 0.373). Anti-apoptotic and pro-apoptotic members of the BCL-2 family (such as BCL-x _L_ (mean PONDR FIT score of 0.330), BCL-2 (mean PONDR FIT score of 0.347), and BCL-W (mean PONDR FIT score of 0.093)) regulate the release of apoptogenic factors from the mitochondria, including cytochrome *c* and an IAP inhibitor SMAC (mean PONDR FIT score of 0.234) ^94, 95^. Caspase-8 mediated cleavage of BID (mean PONDR FIT score of 0.374), a BH3-only member of the BCL-2 family, links the extrinsic and intrinsic pathways ^96^.

Autophagy is induced via the suppression of mTOR (mean PONDR FIT score of 0.089), a sensor of growth factors and nutrient availability, leading to the release of its inhibitory effects on the ULK1 kinase complex (mean PONDR FIT score of 0.546) ^32^. Membrane nucleation requires the class III PI(3)-kinase VPS34 (mean PONDR FIT score of 0.209) and its associated proteins, including Beclin-1 (mean PONDR FIT score of 0.400) ^31^. Two ubiquitin-like conjugation schemes (the ATG5–ATG12 (mean PONDR FIT scores of 0.091 and 0.464, respectively) and LC3-phosphatidylethanolamine (PE) systems (mean PONDR FIT score of 0.306)) mediate the elongation of the autophagosome membrane. At the molecular level, autophagy is mediated by several members of the ATG family that possess various biological functions ^47^. For example, ATG1 (mean PONDR FIT score of 0.546) is a serine/threonine-protein kinase that acts upstream of phosphatidylinositol 3-kinase PIK3C3 to regulate the formation of autophagophores, the precursors of autophagosomes. ATG10 (mean PONDR FIT score of 0.114) and ATG3 (mean PONDR FIT score of 0.452) act as E2-like enzymes responsible for conjugation of ubiquitin-like ATG12 (mean PONDR FIT score of 0.464) to ATG5 (mean PONDR FIT score of 0.091) and ATG8-like proteins (mean PONDR FIT score of 0.306) to PE, respectively, whereas ATG7 (mean PONDR FIT score of 0.064) serves as an E1-like enzyme that facilitates the reaction of conjugation of ATG8-like proteins to PE by forming an E1-E2 complex with ATG3.

Finally, a few words about the major players in necroptosis are given below. One of the better understood necroptosis models is where this pathway is initiated by the ligand-bound tumor necrosis factor receptor 1 (TNFR1) trimers (mean PONDR FIT score of 0.167) ^48^, which recruit multiple proteins such as TNFR-associated death domain (TRADD, mean PONDR FIT score of 0.298), receptor-interacting protein kinase 1 (RIPK1, better known as RIP1, mean PONDR FIT score of 0.276), TRAF2 (mean PONDR FIT score of 0.036), and TRAF5 (mean PONDR FIT score of 0.079). Polyubiquitination of RIP1 triggers the canonical pathway of activation of the transcription factor NF-κB (mean PONDR FIT score of 0.202) which conveys protection against cell death ^97^. However, TNFR1 internalization and RIP1 deubiquitination may lead to the formation of the DISC complex that includes RIP1 (mean PONDR FIT score of 0.276), receptor interacting protein kinase 3 (RIPK3, better known as RIP3, mean PONDR FIT score of 0.431), TRADD (mean PONDR FIT score of 0.298), FAS-associated protein with a death domain (FADD, mean PONDR FIT score of 0.274), and caspase-8 (mean PONDR FIT score of 0.178), which, when activated by the DISC complex, typically initiates apoptosis ^48^. Caspase-8 can be activated not only by TNFR1, but also by other death receptors (such as FAS (mean PONDR FIT score of 0.281) ^98^, TNF-related apoptosis-inducing ligand receptors 1 and 2 (TRAIL-R1, mean PONDR FIT score of 0.323 and TRAIL R2, mean PONDR FIT score of 0.495) ^48^), ultimately leading to the initiation of apoptosis. However, when caspase-8 cannot be activated (i.e., when it is inhibited or depleted), the cell undergoes necroptosis, which is induced by the formation of a necrosis-inducing complex, necrosome, comprised of RIP1 and RIP3 ^99^. This RIP1-RIP3 necrosome generates several pronecroptotic signals, including activation of c-JUN N-terminal kinases 1 and 2 (JNK1, mean PONDR FIT score of 0.155, and JNK2, mean PONDR FIT score of 0.184) that eventually mediates a signaling cascade affecting the iron storage compartment ^99, 100^. Another necroptosis activating pathway is related to the overactivation of poly-ADP-ribose polymerase 1 (PARP1, mean PONDR FIT score of 0.194) leading to the depletion of ATP and NAD, accumulation of poly-ADP-ribose (PAR), and the cytosolic release of apoptosis-inducing factor (AIF, mean PONDR FIT score of 0.162), a protein that is normally secured within the mitochondrial intermembrane space, but upon release from the mitochondria rapidly translocates to the nucleus where it initiates large-scale, caspase-independent DNA fragmentation, leading to further PARP1 activation and subsequent cell death ^48^. Finally, necroptosis is accompanied by the release of high-mobility group box 1 protein (HMGB1, mean PONDR FIT score of 0.774), which is both a nuclear factor (which serves as an architectural chromatin-binding factor that bends DNA and promotes protein assembly on specific DNA targets) and a secreted protein serving as a potent mediator of inflammation ^101^. This HMGB1 release is a diffusible signal of necroptosis, which can be used as a cue to nearby cells ^102^.

### Intrinsic disorder of the major regulators of the p53-mediated apoptotic signaling pathways

Figure 2 zooms into the bottom left corner of Figure 1 and shows the p53-mediated apoptotic signaling pathways in more detail. At the center of this signaling pathways is the tumor-related protein p53 (mean PONDR FIT score of 0.519). p53 is a crucial transcription factor, its activation in response to genotoxic or cellular stresses is known to induce or inhibit more than 150 genes ^103, 104^. Some of the p53 targets are important players in apoptosis, growth arrest, or senescence pathways ^62, 105– 107^. Since p53 is an important regulator of various cellular processes (including apoptosis), it has a short life-time and is normally maintained at low levels in unstressed mammalian cells via continuous ubiquitination and subsequent proteasomal degradation. Normally, ubiquitination of non-phosphorylated p53 is driven by the mouse double minute-2 ubiquitin ligase (Mdm2, mean PONDR FIT score of 0.550), which is capable of targeting p53 for degradation ^108^. This process is regulated via negative feedback between the p53 and Akt pathways ^109^. Here, Akt (mean PONDR FIT score of 0.173) is activated in cells exposed to diverse stimuli such as hormones, growth factors, and extracellular matrix components ^110^.

In humans, there is a myriad of growth factors that are able to stimulate cellular growth, proliferation, and cellular differentiation, and some of them can also be involved in apoptosis ^111^. Since growth factor proteins typically occur as members of rather large families of related proteins, the real number of the representatives of this protein class is noticeably larger. For example, there are 23 members in the fibroblast growth factor family (the comprehensive analysis of this very interesting class of proteins is outside the scope of this study). One of the characteristic members of this family of proteins is fibroblast growth factor 1 (FGF1, mean PONDR FIT score of 0.291) that is involved in the regulation of apoptosis. The binding of growth factors to growth factor receptors (e.g., to the fibroblast growth factor receptor 2, FGFR2, mean PONDR FIT score of 0.248) leads to their dimerization and activates their tyrosine kinase activity, causing autophosphorylation of tyrosine residues on the intracellular domain of the receptor. This recruits the p85 regulatory subunit of the phosphoinositol 3-kinase (PI3K p85, mean PONDR FIT score of 0.413) via its SH2 domain, mediating the association of the PI3K p110 catalytic unit (mean PONDR FIT score of 0.164) to the plasma membrane ^109, 110^. Activated PI3K phosphorylates membrane-bound PIP2 (phosphatidylinositol-3,4-bisphosphate) to generate PIP3 (phosphatidylinositol-3,4,5-triphosphate) which then binds to Akt via its PH domain, eventually leading to Akt activation ^109, 110^. Activated Akt then specifically phosphorylates Mdm2 at position Ser166 which promotes cell survival via Mdm2-mediated inhibition and destruction of p53 ^109^. This cell survival pathway is blocked under stress conditions where Akt is degraded and PI3K is inhibited via phosphatase PTEN (phosphatase and tensin homolog, mean PONDR FIT score of 0.280). The multifarious roles of intrinsic disorder on the functions of PTEN and its interactomes were covered in a recent study ^112^. Particularly, it was shown that PTEN possesses an IDPR at its C-terminus. This disordered C-tail, with its set of MoRFs, conserved eukaryotic linear motifs, and numerous sites of posttranslational modifications, plays a crucial role in a multitude of PTEN-based protein-protein interactions ^112^. Since many proteins in the primary and secondary interactomes of PTEN possessed significant amount of intrinsic disorder, and since many of these PTEN-interacting IDPs were cancer-related, it has been concluded that PTEN represents a pliable, intrinsically disordered, cancer-related hub located within a flexible network of cancer-related IDPs ^112^.

One of the most important mechanisms defining the ability of p53 to possess various regulatory functions consists of the precise control of its posttranslational modifications (PTMs), such as phosphorylation, acetylation, sumoylation, and ubiquitination. For example, in response to DNA damage, the cell cycle checkpoint kinases 1 and 2 (Chk1 and Chk2, mean PONDR FIT scores of 0.252 and 0.343, respectively) become activated by ataxia-telangiectasia and Rad3 related kinase (ATR, mean PONDR FIT score of 0.170) and ataxia-telangiectasia mutated kinase (ATM, mean PONDR FIT score of 0.194), respectively. Activated Chk1 and Chk2 phosphorylate p53 at multiple positions, with Chk1 phosphorylating multiple sites within the intrinsically disordered C-terminus of p53 ^113^ and Chk2 primarily targeting the intrinsically disordered N-terminal region of p53 ^114^. It is important to note that the Chk2-induced p53 phosphorylation at Ser20 and ATM-driven p53 phosphorylation at position Ser15 stabilize p53 and promote accumulation of activated p53 in the nucleus ^114^. Activated p53 binds to the consensus p53 response elements of several genes, particularly those encoding proteins from both the extrinsic and intrinsic apoptotic pathways ^103, 104^.

In the intrinsic apoptotic pathway, p53 induces the expression of several members of the BCL-2 family (whose family members are classified on the basis of their structural similarity to the BH (BCL-2 homology) domains (BH1, BH2, BH3, and BH4) and a transmembrane domain), such as multidomain BCL-2 family member BAX (mean PONDR FIT score of 0.310) ^115^, together with the BH3-only BCL-2 family members PUMA (p53-upregulated modulator of apoptosis, mean PONDR FIT score of 0.896) ^116^, NOXA (mean PONDR FIT score of 0.578) ^117^, and BID (BH3 interacting domain death agonist, mean PONDR FIT score of 0.374) ^118^.

Under stress conditions, homodimeric BAX undergoes a conformation change that causes translocation to the mitochondrial membrane and formation of higher BAX oligomers. Mulitmerization of BAX and subsequent translocation to mitochondria are promoted by a family of PUMA proteins produced via the alternative splicing of a p53 transactivation target gene, *PUMA* ^116^. Another p53 target gene, *Noxa* encodes a BH3-only protein, which contributes to the p53-controlled apoptosis in a similar manner to PUMA and BAX. Also, there is another p53 target gene encoding for the p53-regulated apoptosis-inducing protein 1 (p53AIP1, mean PONDR FIT score of 0.492), which is located in the mitochondrial membrane and is directly involved in the p53-dependent mitochondrial apoptosis ^119^. Translocation of BAX to the mitochondrion membrane initiates the release of cytochrome *c* (mean PONDR FIT score of 0.327). At the next stage, the apoptosome complex comprising of cytochrome *c*, the oligomeric form of the apoptotic protease-activating factor-1 (APAF1, mean PONDR FIT score of 0.153), and procaspase-9 (mean PONDR FIT score of 0.271) is formed. This complex then activates caspase-9, which promotes the activation of caspase-3 (mean PONDR FIT score of 0.303), caspase-6 (mean PONDR FIT score of 0.233), and caspase-7 (mean PONDR FIT score of 0.221), leading to the subsequent cleavage of vital death substrates and culminating in cell death.

The p53-stimulated extrinsic apoptotic pathway relies on the engagement of the specific cell-surface death receptors from the TNDR family needed for the activation of specific caspases, such as caspase-8 (mean PONDR FIT score of 0.178) and caspase-3 (mean PONDR FIT score of 0.303), leading to the eventual induction of apoptosis. Among the most common death receptors involved in extrinsic apoptosis are TNDR superfamily member 6 or apoptosis-mediating surface antigen FAS (FAS, CD95, or Apo-1, mean PONDR FIT score of 0.281), death receptor-5 (DR5, mean PONDR FIT score of 0.437), and p53 apoptosis effector related to PMP-22 (PERP, mean PONDR FIT score of 0.215). FAS is activated by binding its ligand, FasL (mean PONDR FIT score of 0.394), which is a homotrimeric protein expressed predominantly by T-cells that causes FAS oligomerization on binding. FAS oligomerization is accompanied by the clustering of their death domains and the formation of the death-inducing signaling complex (or DISC) comprising of FAS (mean PONDR FIT score of 0.281), FADD (mean PONDR FIT score of 0.274), death ligand (mean PONDR FIT score of 0.448), and procaspase-8 (mean PONDR FIT score of 0.301) that binds to the death effector domain of FADD via a homologous motif ^120, 121^.

In relation to the p53-mediated extrinsic apoptotic pathway, p53 plays a dual role in FAS-induced apoptosis. Firstly, in response to the gamma-irradiation of specific tissues, FAS mRNA expression is induced by p53 binding to the specific elements within the promoter and first intron of the *FAS* gene. Secondly, overexpressed p53 can rapidly sensitize cells to FAS-induced apoptosis before the transcription-dependent effect operates, by increasing FAS levels at the cell surface via the promotion of the FAS receptor trafficking from the Golgi apparatus ^120, 121^.

The second member of the TNDR family induced by p53 is the death-domain-containing receptor for TNF-related apoptosis-inducing ligand (TRAIL, mean PONDR FIT score of 0.349), killer or death receptor-5 (DR5, mean PONDR FIT score of 0.437). In response to DNA damage, DR5 is induced by p53 in a cell type specific manner, initiating apoptosis through caspase-8. Finally, the p53 apoptosis effector related to PMP-22 (PERP, mean PONDR FIT score of 0.215) is assumed to serve as a direct p53 target, since the *PERP* promoter contains a p53-responsive element and since PERP production is induced in response to DNA damage. PERP is a member of the PMP-22/Gas family of tetraspan transmembrane proteins implicated in the regulation of cell growth. Although the precise role of PERP in p53-mediated apoptosis is yet to be understood, this protein can be involved in at least three potential mechanisms. First, PERP could serve as a cell death receptor that receives specific apoptosis-initiating signals. Second, due to its sequence similarity to the calcium channel, PERP could possess channel or pore activity and play a role in apoptosis activation by passing through some crucial molecules. Third, PERP might directly affect some regulators of apoptotic machinery, such as BAX (mean PONDR FIT score of 0.310) or BCL-2 (mean PONDR FIT score of 0.347), or even directly act on some crucial apoptotic effectors, such as the caspases ^122, 123^.

**Figure 3.**
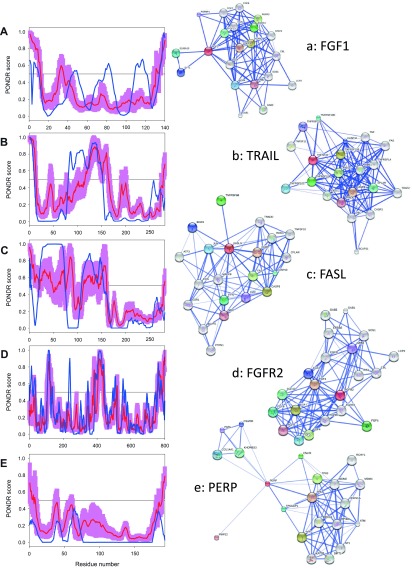
Intrinsic disorder (upper characters) of some human proteins involved in the p53-mediated apoptotic signaling pathways and STRING analysis (lower characters) of their interactomes: group I. **A**. and **a**. Growth/survival factor (Fibroblast growth factor 1, UniProt ID: P05230); **B**. and **b**. TRAIL (UniProt ID: P50591); **C**. and **c**. FasL (UniProt ID: P48023); **D**. and **d**. Growth factor receptor (Fibroblast growth factor receptor 2, UniProt ID: P21802); **E**. and **e**. PERP (UniProt ID: Q96FX8). Intrinsic disorder propensity was evaluated by PONDR-FIT (red curves and pink shadow) and PONDR ^®^ VLXT (blue curves). The shadow around PONDR-FIT curves represents distribution of statistical errors. STRING database is the online database resource search tool for the Retrieval of Interacting Genes, which provides both experimental and predicted interaction information ^92^. For each protein, STRING produces the network of predicted associations for a particular group of proteins. The network nodes are proteins. The edges represent the functional associations evaluated based on experiments, search of databases, and text mining. The thickness of edges is proportional to the confidence level ^92^.

To further delve into the abundance and functional roles of intrinsic disorder in the proteins discussed above from the intrinsic and extrinsic apoptotic pathways mediated by p53, Figure 3– Figure 8 represent disorder profiles plotted using the results of the analysis of these proteins by PONDR-FIT and PONDR ^®^ VLXT disorder predictors, and also shows the results of the functional analysis of these proteins using the STRING database ^92^. Figure 3– Figure 8 show that all the members of the p53-controlled intrinsic and extrinsic apoptosis signaling pathways possess noticeable amounts of intrinsic disorder and are involved in multiple interactions with other apoptosis-related proteins (and also with many other proteins not directly related to the apoptosis), which are often predicted to be disordered. In protein-protein interaction networks, the highly connected nodes (proteins) with many edges (interactions) are known as hubs. Earlier analysis revealed that protein intrinsic disorder is crucial for the binding promiscuity of hub proteins ^37, 124^. In fact, it has been shown that although hub proteins can be entirely disordered or contain long IDPRs or be highly structured, they utilize intrinsic disorder for protein-protein interactions via at least two mechanisms: one disordered region binding to many partners or many disordered region binding to one partner ^37, 124– 132^. Therefore, in agreement with these previous studies, our analysis revealed that the major players of the p53-modulated apoptosis can be considered as disordered hub proteins.

**Figure 4.**
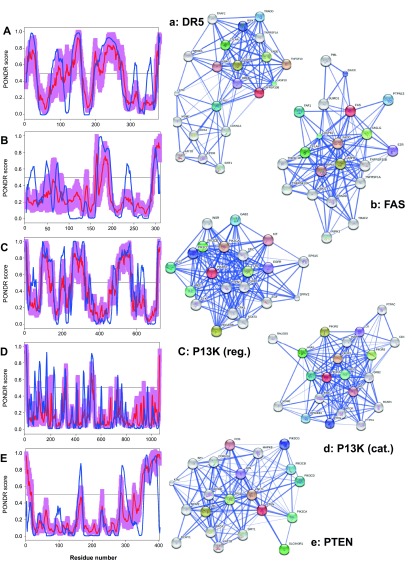
Intrinsic disorder (upper characters) of some human proteins involved in the p53-mediated apoptotic signaling pathways and STRING analysis (lower characters) of their interactomes: group II. **A**. and **a**. DR5 (UniProt ID: O14763); **B**. and **b**. FAS (UniProt ID: P25445); **C**. and **c**. PI3K regulatory subunit (UniProt ID: O00459); **D**. and **d**. PI3K catalytic subunit (UniProt ID: P42336); **E**. and **e**. PTEN (UniProt ID: P60484). Intrinsic disorder propensity was evaluated by PONDR-FIT (red curves and pink shadow) and PONDR ^®^ VLXT (blue curves). The shadow around PONDR-FIT curves represents distribution of statistical errors. STRING database is the online database resource search tool for the Retrieval of Interacting Genes, which provides both experimental and predicted interaction information ^92^. For each protein, STRING produces the network of predicted associations for a particular group of proteins. The network nodes are proteins. The edges represent the functional associations evaluated based on experiments, search of databases, and text mining. The thickness of edges is proportional to the confidence level ^92^.

**Figure 5.**
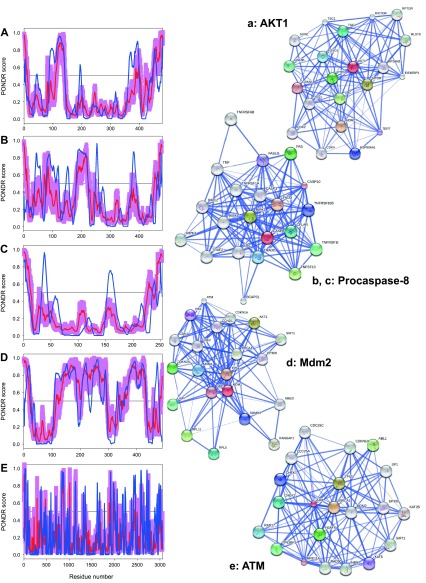
Intrinsic disorder (upper characters) of some human proteins involved in the p53-mediated apoptotic signaling pathways and STRING analysis (lower characters) of their interactomes: group III. **A**. and **a**. AKT1 (UniProt ID: P31749); **B**. and **b**. Procaspase-8 (UniProt ID: Q14790); **C**. and **c**. Caspase-8 (residues 217–374 and 385–479; ID: Q14790); **D**. and **d**. Mdm2 (UniProt ID: Q00987); **E**. and **e**. ATM (UniProt ID: Q13315). Intrinsic disorder propensity was evaluated by PONDR-FIT (red curves and pink shadow) and PONDR ^®^ VLXT (blue curves). The shadow around PONDR-FIT curves represents distribution of statistical errors. STRING database is the online database resource search tool for the Retrieval of Interacting Genes, which provides both experimental and predicted interaction information ^92^. For each protein, STRING produces the network of predicted associations for a particular group of proteins. The network nodes are proteins. The edges represent the functional associations evaluated based on experiments, search of databases, and text mining. The thickness of edges is proportional to the confidence level ^92^.

**Figure 6.**
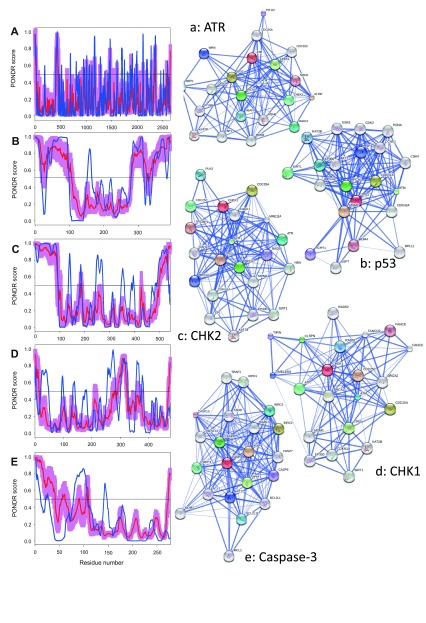
Intrinsic disorder (upper characters) of some human proteins involved in the p53-mediated apoptotic signaling pathways and STRING analysis (lower characters) of their interactomes: group IV. **A**. and **a**. ATR (UniProt ID: Q13535); **B**. and **b**. p53 (UniProt ID: P04637); **C**. and **c**. CHK2 (UniProt ID: O96017); **D**. and **d**. CHK1 (UniProt ID: O14757); **E**. and **e**. Caspase-3 (UniProt ID: P42574). Intrinsic disorder propensity was evaluated by PONDR-FIT (red curves and pink shadow) and PONDR ^®^ VLXT (blue curves). The shadow around PONDR-FIT curves represents distribution of statistical errors. STRING database is the online database resource search tool for the Retrieval of Interacting Genes, which provides both experimental and predicted interaction information ^92^. For each protein, STRING produces the network of predicted associations for a particular group of proteins. The network nodes are proteins. The edges represent the functional associations evaluated based on experiments, search of databases, and text mining. The thickness of edges is proportional to the confidence level ^92^.

**Figure 7.**
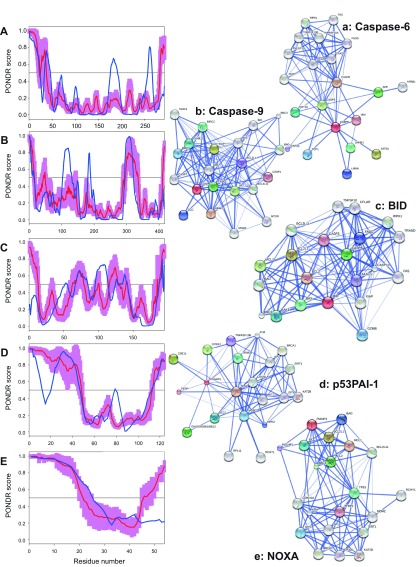
Intrinsic disorder (upper characters) of some human proteins involved in the p53-mediated apoptotic signaling pathways and STRING analysis (lower characters) of their interactomes: group V. **A**. and **a**. Caspase-6 (UniProt ID: P55212); **B**. and **b**. Caspase-9 (UniProt ID: P55211); **C**. and **c**. BID (UniProt ID: P55957); **D**. and **d**. p53PAI-1 (UniProt ID: Q9HCN2); **E**. and **e**. NOXA (UniProt ID: Q13794). Intrinsic disorder propensity was evaluated by PONDR-FIT (red curves and pink shadow) and PONDR ^®^ VLXT (blue curves). The shadow around PONDR-FIT curves represents distribution of statistical errors. STRING database is the online database resource search tool for the Retrieval of Interacting Genes, which provides both experimental and predicted interaction information ^92^. For each protein, STRING produces the network of predicted associations for a particular group of proteins. The network nodes are proteins. The edges represent the functional associations evaluated based on experiments, search of databases, and text mining. The thickness of edges is proportional to the confidence level ^92^.

**Figure 8.**
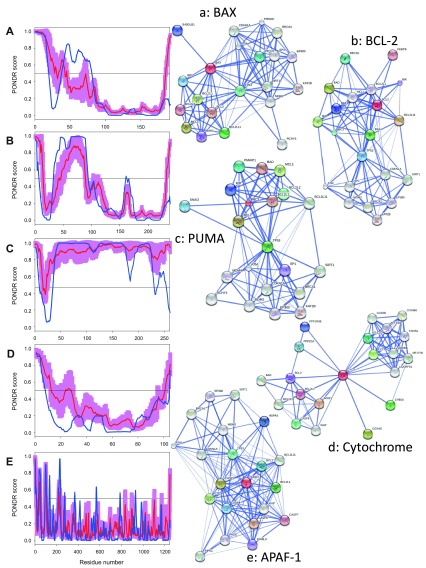
Intrinsic disorder (upper characters) of some human proteins involved in the p53-mediated apoptotic signaling pathways and STRING analysis (lower characters) of their interactomes: group VI. **A**. and **a**. BAX (UniProt ID: Q07812); **B**. and **b**. BCL-2 (UniProt ID: P10415); **C**. and **c**. PUMA (UniProt ID: Q96PG8); **D**. and **d**. Cytochrome *c* (UniProt ID: P99999); **E**. and **e**. APAF-1 (UniProt ID: O14727). Intrinsic disorder propensity was evaluated by PONDR-FIT (red curves and pink shadow) and PONDR ^®^ VLXT (blue curves). The shadow around PONDR-FIT curves represents distribution of statistical errors. STRING database is the online database resource search tool for the Retrieval of Interacting Genes, which provides both experimental and predicted interaction information ^92^. For each protein, STRING produces the network of predicted associations for a particular group of proteins. The network nodes are proteins. The edges represent the functional associations evaluated based on experiments, search of databases, and text mining. The thickness of edges is proportional to the confidence level ^92^.

**Figure 9.**
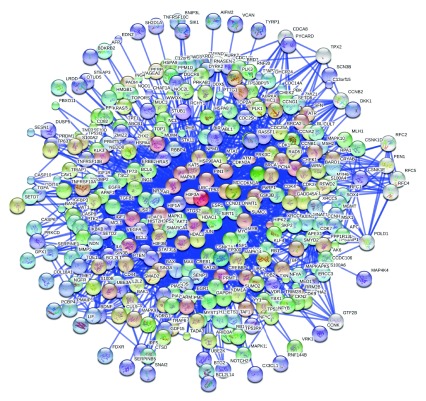
STRING-based extended interactome of p53. This network was built by setting the number of interactors to 500 and the STRING confidence level of 0.900. Therefore, this network includes only partners whose interactions are predicted with the highest confidence level.

One should remember, however, that the interactomes of various proteins involved in the regulation and execution of p53-mesdiated apoptosis are in fact essentially larger than those shown in Figure 3– Figure 8. In fact, to generate the corresponding plots, the standard set of parameters was used in the STRING database, namely, Active Prediction Methods: Neighborhood, gene fusion, co-occurrence, co-expression, experiments, databases, and textmining; Required confidence (score): medium confidence (0.400); Interactors shown: no more than 20 interactors. Figure 9 illustrates how the predicted interactome of p53 changes when the number of interactors is increased to 500 (which is the highest allowed value) and the STRING confidence level is boosted to the value of 0.900. Figure 9 gives further support to the notion that, being an intrinsically disordered hub, p53 is located at the middle of a large and well-developed network of protein-protein interactions, serving as a crucial linker that connects multiple important pathways. Analysis of the disorder status in the 500 proteins known to interact with p53 is outside the scope of this study and will be reported elsewhere.

### Looking at intrinsic disorder from the protein structure angle

**Figure 10.**
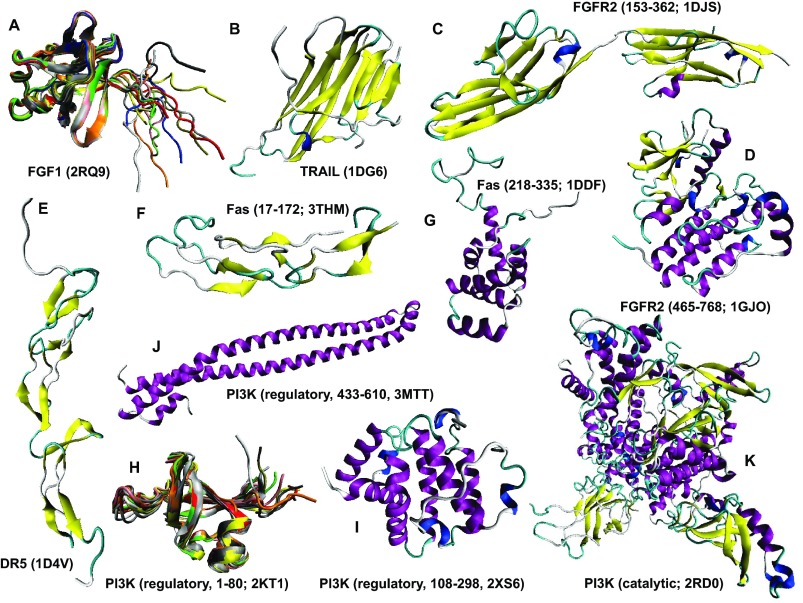
Structural characterization of human proteins involved in the p53-mediated apoptotic signaling pathways. **A**. Fibroblast growth factor 1 (PDB ID: 2RQ9); **B**. TRAIL (PDB ID: 1DG6); **C**. Fibroblast growth factor receptor 2 (residues 153–362; PDB ID: 1DJS); **D**. Fibroblast growth factor receptor 2 (residues 465–768; PDB ID: 1GJO); **E**. DR5 (PDB ID: 1D4V); **F**. FAS (Residues 17–172; PDB ID: 3THM); **G**. FAS (Residues 218–335; PDB ID: 1DDF); **H**. PI3K (regulatory subunit, 1–80; PDB ID: 2KT1); **I**. PI3K (regulatory 108–298; PDB ID: 2XS6); **J**. PI3K (regulatory subunit, 433–610; PDB ID: 3MTT); **K**. PI3K (catalytic subunit; PDB ID: 2RD0).

Figure 10– Figure 14 represent structural information available in protein databank (PDB) ^133^ for many of the human proteins associated with the p53-mediated apoptotic signaling pathways. In fact, at least partial structural information is available for 23 of the 29 human proteins discussed above. The lack of complete structural information for these proteins can be explained, at least in part, by the abundance of intrinsic disorder which hinders crystallization. In fact, two of the structurally uncharacterized proteins, p53PAI-1 and PUMA (see Figure 8), are among the most disordered members of the analyzed group, possessing average disorder scores of 0.492 and 0.896, respectively. Figure 3C shows that a significant part of FasL is predicted to be highly disordered. Although ATM and ATR are mostly ordered (see Figure 5 and Figure 6), these are large kinases that possess a noticeable amount of relatively long disordered regions. Finally, it is expected that crystallization of PERP ( Figure 3E) would be a challenging task since this p53 apoptosis effector is a typical transmembrane protein with four transmembrane domains, and transmembrane proteins are known to be rather tough targets for structural characterization.

**Figure 11.**
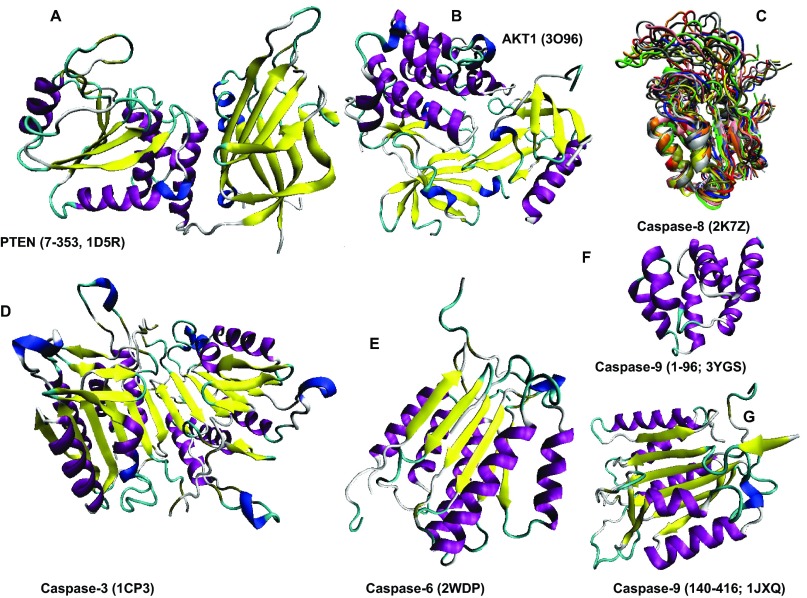
Structural characterization of human proteins involved in the p53-mediated apoptotic signaling pathways: group I. **A**. PTEN (PDB ID: 1D5R); **B**. AKT1 (PDB ID: 3O96); **C**. Caspase-8 (PDB ID: 2K7Z); **D**. Caspase-3 (PDB ID: 1CP3); **E**. Caspase-6 (PDB ID: 2WDP); **F**. Caspase-9 (Residues 1–96; PDB ID: 3YGS); **G**. Caspase-9 (Residues 140–416; PDB ID: 1JXQ).

**Figure 12.**
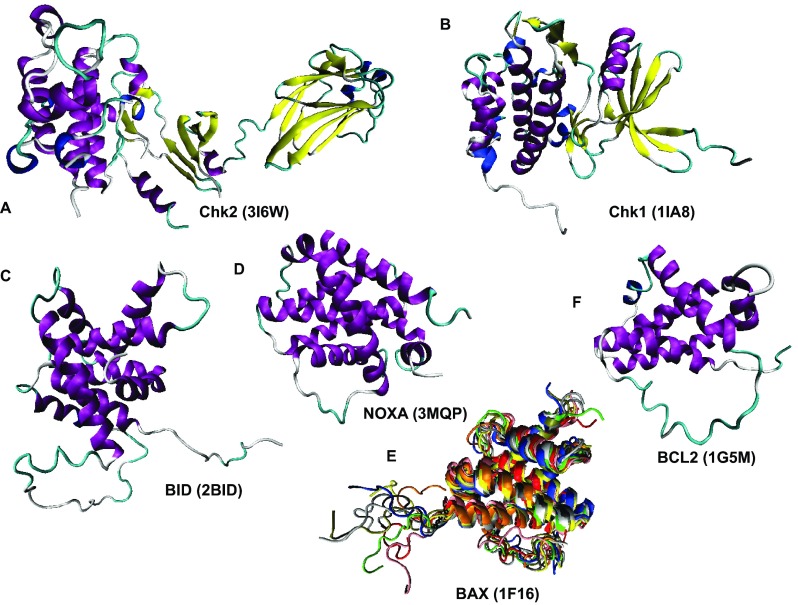
Structural characterization of human proteins involved in the p53-mediated apoptotic signaling pathways: group II. **A**. CHK2 (PDB ID: 3I6W); **B**. CHK1 (PDB ID: 1IA8); **C**. BID (PDB ID: 2BID); **D**. NOXA (PDB ID: 3MPQ); **E**. BAX (PDB ID: 1F16); **F**. BCL-2 (PDB ID: 1GM5).

**Figure 13.**
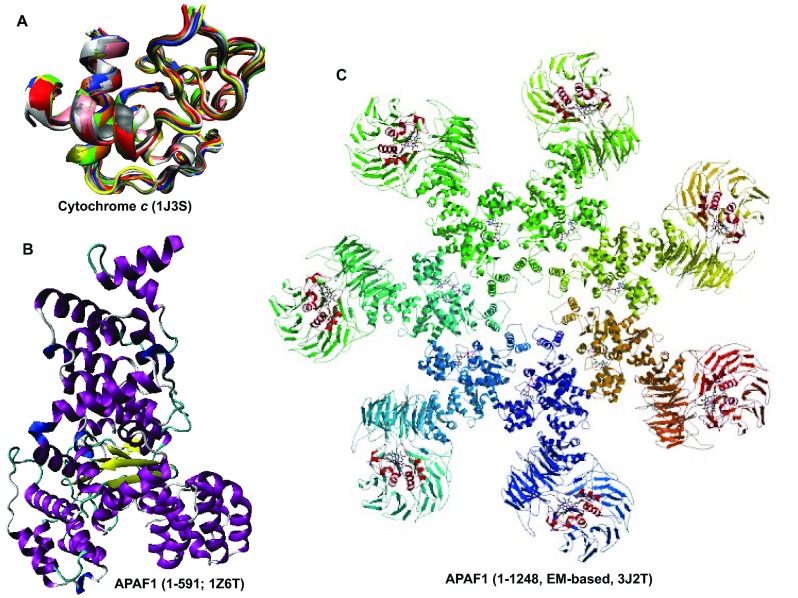
Structural characterization of human proteins involved in the p53-mediated apoptotic signaling pathways: group III. **A**. Cytochrome *c* (PDB ID: 1J3S); **B**. APAF-1 (Residues 1–591; PDB ID: 1Z6T); **C**. APAF-1 (PDB ID: 3J2T).

**Figure 14.**
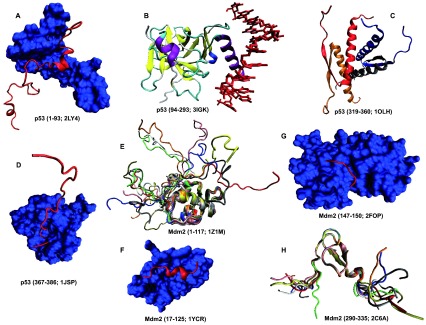
Structural characterization of human proteins involved in the p53-mediated apoptotic signaling pathways: group IV. **A**. Residues 1–93 of p53 (red ribbon) in a complex with the bromodomain of CREB-binding protein (blue surface) (PDB ID: 2LY4); **B**. Residues 94–293 of p53 in a complex with DNA (red bonds) (PDB ID: 3IGK); **C**. Residues 319–360 of p53 in a homotetrameric complex (PDB ID: 1OLH); **D**. Residues 367–386 of p53 (red ribbon) in a complex with the bromodomain of CREB-binding protein (blue surface) (PDB ID: 1JSP); **E**. Residues 1–117 of MDM2 (PDB ID: 1Z1M); **F**. Residues 17–125 of MDM2 (blue surface) in a complex with the transactivation domain of p53 (residues 15–29, red ribbon) (PDB ID: 1YCR); **G**. Residues 147–150 of Mdm2 (red ribbon) in a complex with the N-terminal domain of HAUSP/USP7 (blue surface) (PDB ID: 2FOP); **H**. Residues 290–335 of Mdm2 (PDB ID: 2C6A).

Analysis of structures shown in Figure 10– Figure 14 and obtained by protein crystallization and NMR studies suggests that many of the human proteins associated with the p53-mediated apoptotic signaling pathways possess noticeable conformational flexibility. This is evidenced by the following facts:

a) Crystallization of a full length protein was often impossible and many structures shown in Figure 10– Figure 14 correspond to the protein domains and short fragments;

b) Proteins of interest and their fragments were frequently co-crystallized in the complexes with specific binding partners;

c) Proteins or protein fragments often do not possess globular shape suggesting that the resulting structures are induced by binding to specific partners (e.g., Figure 10E, 10F, 10J, Figure 14A, 14C, 14D, 14F, and 14G);

d) The NMR structures are characterized by the "fuzzy" appearance typical of the highly dynamic structures (e.g., see Figure 10A, 10H, Figure 11C, Figure 12E, Figure 13A, Figure 14E, and 14H);

e) Many proteins contain long structureless tails and loops (e.g., see Figure 10B, 10G, Figure 11A, 11E, 11G, Figure 12B, 12C, 12D, and 12F);

f) Even the determined structures of many proteins contain structurally undefined regions known as regions of missing electron density (see Table 1). The flexibility or disorder of a protein region determines the failure of this region to scatter X-ray coherently and therefore explains the invisibility of this region in a crystal structure ^134^.

**Table 1.** Regions of missing electron density in human proteins involved in the p53-mediated apoptotic pathways.

Protein name	PDB ID	Regions of missing electron density
TRAIL	1DG6	91–119, 131–141, 195–196
Fibroblast growth factor receptor 2	1DJS 1GJO	297–306 453–466, 504–506, 581–595, 766–768
FAS	3THM	1–35, 92–93, 122–164
PI3K (regulatory subunit)	2XS6	85–108, 202–203, 257–274, 295–298
PI3K (catalytic subunit)	2RD0	1–7, 307–324, 415–423, 506–527, 941–950, 1051–1068
PTEN	1D5R	7–13, 282–285, 309–312, 352–353
AKT	3O96	46–48, 89–91, 114–144, 189–198, 299–312, 430–443
Caspase-3	1CP3	1–34, 174–184
Caspase-6	2WDP	1–30, 166–199, 262–266
Caspase-9	1JXQ	139–147, 296–297, 404–409
Chk2	3I6W	70–91, 228–232, 254–267, 370–401, 502–512
Chk1	1IA8	1, 46–47, 277–289
BCL2	1GM5	1–2
APAF-1	1Z6T	95–104, 587–591

All these observations provide strong support to the notions that intrinsic disorder is abundant in human proteins associated with the p53-mediated apoptotic signaling pathways and that disordered regions are frequently used for protein-protein interactions needed for the efficient control and regulation of the mentioned pathways.

### Abundance of the disorder-based binding sites in human proteins associated with the p53-mediated apoptotic signaling pathways

IDPs and IDPRs are commonly engaged in protein-protein interactions and play crucial roles in molecular recognition ^2, 8, 10, 30, 84, 135– 143^. In the process of their function and interaction with their binding partners, many IDPs and IDPRs are known to undergo at least partial disorder-to-order transitions, and these conformational transitions are crucial for recognition, regulation, and signaling ^4, 8, 84, 143– 149^. Based on the specific features and patterns in the per-residue disorder propensity curves calculated by the PONDR ^®^ VLXT tool combined with the analysis of several characteristic sequence-based parameters, short disordered regions capable of undergoing the disorder-to-order transitions on binding can be found by specific predictors ^84, 85^. These disorder-based binding regions are known as α-helix forming MoRFs (α-MoRFs). Table 2 shows that α-MoRFs are common in human proteins involved in p53-mediated apoptotic pathways, and that some long disordered proteins have multiple α-MoRFs, which likely serve as promiscuous binders interacting with multiple partners.

**Table 2.** Abundance of disorder-based binding sites in proteins involved in the p53-mediated apoptotic pathways.

Protein name	UniProt ID	MoRF	AIBS ^a^	Experiment	Reference
Fibroblast growth factor 1	P05230	-	28–39	24–27 (NLS) ^b^	UniProt
TRAIL	P50591	-	*179–189*		
FasL	P48023	4–21, 83–101, 133–150	9–19, *77–110*	81–102	UniProt
Fibroblast growth factor receptor 2	P21802	-	*7–12*, 117–126, 171–178, *380–396*, 488–498, 756–761, *808–812*	764–778	[ 153]
PERP	Q96FX8	-	*1–15*, *177–190*	-	-
DR5	O14763	37–55, 100–118, 165–182	6–15, 34–57, *214–247*, 272–279, 302–312, 339–352	-	-
FAS	P25445		*1–15*, *177–190*	230–254	UniProt
PI3K regulatory subunit	O00459	487–504, 565–582	29–43, 70–84, 109–118, 122–136, 169–183, 218–231, 241–256, 277–298, 312–320, 325–335, 395–400, *484–488*, 574–581	-	-
PTEN	P60484	386–403	*316–320*, 336–350, *376–380*, 397–403	391–403	UniProt
AKT1	P31749	414–431	*56–60*, 73–84, *151–155*, *406–414*, 441–447, 471–480	-	-
Procaspase-8	Q14790	228–245	233–242, *399–404*, 421–426, *440–444*, 472–479	-	-
Mdm2	Q00987	187–205, 228–245, 270–287, 338–356	100–108, 189–208, 235–261, 273–284, 296–306, 322–329, 359–367, 399–409	145–150, 179–185, 190–202, 223–232	[ 154], UniProt
ATM	Q13315	1–18, 1992–2009	*1–12*, *60–64*, 896–907, 2558–2563, *2622–2632*, *2950–2957*	1373–1382	UniProt
ATR	Q13535	-	*2634–2638*	-	-
p53	P04637	17–35, 321–338, 374–398	11–57, 106–115, 132–141, 232–239, 251–258, 265–277, 322–355, 363–387	13–29, 1–39, 33–60, 241–288, 273–280, 326–356, 359–363, 367–388	UniProt, [ 155– 159]
CHK2	O96017	526–543	1–26, 35–41, 58–74, 94–110, *415–419*, 535–543	63–73	[ 160]
CHK1	O14757	285–302, 333–350	*240–244*, 294–300, 334–342	-	-
Caspase-3	P42574	115–133	41–53, 114–119	-	-
Caspase-6	P55212	19–37	-	-	-
Caspase-9	P55211	-	71–77, 281–286	-	-
BID	P55957	-	*17–27*, *86–90*, 105–111, 148–157	76–109	[ 161]
p53PAI-1	Q9HCN2	8–25	8–17	-	-
NOXA	Q13794	-	*38–54*	19–43	UniProt
BAX	Q07812	21–38	18–34	13–19, 48–81	[ 162, 163]
BCL-2	P10415	-	13–32, 46–51	-	-
PUMA	Q96PG8	12–30, 174–192, 231–248	1–11, 15–29, 61–80, 85–99, 102–119, 123–261	-	-
Cytochrome *c*	P99999	-	9–18	-	-

^a^ ANCHOR-indicated binding site. Numbers shown in italic correspond to regions (longer than 5 residues) that were predicted as potential binding site by ANCHOR, but filtered due to low IUPred disorder scores (below 0.1) ^86, 87^.

^b^ NLS, Nuclear localization signal.

MoRF, Molecular recognition feature.

Another way to find potential disorder-based binding sites is based on looking for residues in disordered regions that cannot form enough favorable intra-chain interactions to fold on their own, but which are likely to gain stabilizing energy by interacting with a globular protein partner ^86, 87^. This approach is based on using the ANCHOR algorithm ^86, 87^ to find disordered but foldable binding regions, ANCHOR-identified binding sites (AIBSs). ANCHOR relies on the pairwise energy estimation approach that is the basis for IUPred, a general disorder prediction method ^82^, whereas MoRF identifiers rely on specific patterns in the per-residue disorder propensity curves calculated by the PONDR ^®^ VLXT ^78^. Since methodologically and logistically ANCHOR and MoRF identifiers are very different, the application of both tools provides complementary information. Table 2 shows that AIBSs are very common among the human proteins involved in the p53-mediated apoptotic pathways. In fact, the majority of these proteins possess more than one AIBS, and many of them have multiple AIBSs. Furthermore, there is typically a reasonable agreement between the outputs of these two tools.

Finally, Table 2 lists some of the experimentally validated binding sites found in the human proteins involved in the p53-mediated apoptotic pathways. These potential binding sites were typically found based on the analysis of the reported x-ray crystallography or NMR data on structural characterization of a fragment of a target protein bound to its partner(s). Table 2 shows that the majority of the experimentally found binding sites are correctly predicted by at least one algorithm. This observation provides indirect support to the validity of the results of the computational analysis and to the conclusion that the major function of many IDPRs in the human proteins involved in the p53-mediated apoptotic pathways is related to regulation and control via the protein-protein interactions.

## Concluding remarks

In conclusion, the results presented in this paper clearly show that many of the human proteins involved in regulation and execution of three major programmed cell death pathways: apoptosis, autophagy, and necroptosis, as well as human proteins that are involved in the p53-mediated apoptotic signaling pathways possess substantial amounts of intrinsic disorder. Since functional repertoires of ordered and disordered proteins are very different, finding high prevalence of disorder in PCD-related proteins clearly indicates that careful consideration of this important feature is absolutely critical for better understanding the structure and conformational behavior of these proteins, their promiscuity, and molecular mechanisms of their functions, regulation, and control. In agreement with this idea, we show here that most major players in three major PCD pathways and in particular proteins related to the p53-mediated apoptosis have relative high levels of intrinsic disorder and that there is a positive relationship between protein disorder and the number of interactions a protein has. Furthermore, many of these proteins contain multiple disorder-based protein interaction sites. These observations suggest that intrinsic disorder might be intimately related to all the aspects of activity of these proteins and plays indispensible roles in their functional interactions.

## List of abbreviations

AIBS, ANCHOR-indicated binding site

AIF, apoptosis inducing factor

APAF1, apoptotic protease-activating factor 1

ATM, ataxia-telangiectasia mutated kinase

ATR, ataxia-telangiectasia and Rad3 related kinase

BID, BH3-interacting domain death agonist

Chk1, cell cycle checkpoint kinase 1

Chk2, cell cycle checkpoint kinase 2

DAPK, death-associated protein kinase

DISC, death-inducing signaling complex

DR5, death receptor-5

FADD, FAS-associated death domain protein

FasL, FAS ligand

FGFR2, fibroblast growth factor receptor 2

GFG1, fibroblast growth factor 1

HMGN1, high-mobility group box 1

IAP, inhibitor of apoptosis protein

IDP, intrinsically disordered protein

IDPR, intrinsically disordered protein region

JNK, c-Jun N-terminal kinase

Mdm2, mouse double minute-2 ubiquitin ligase

MoRF, molecular recognition feature

mTOR, mammalian target of rapamycin kinase

p53AIP1, p53-regulated apoptosis-inducing protein 1

PAR, polyADP-ribose

PARP1, poly-ADP-ribose polymerase 1

PE, phosphatidylethanolamine

PERP, p53 apoptosis effector related to PMP-22

PCD, programmed cell death

PDB, protein databank

PI3K, phosphoinositol 3-kinase

PIP2, phosphatidylinositol-3,4-bisphosphate

PTEN, Phosphatidylinositol 3,4,5-trisphosphate 3-phosphatase and dual-specificity protein phosphatase PTEN

PTM, posttranslational modification

RIPK1, receptor-interacting protein kinase 1

SMAC, second mitochondria-derived activator of caspase

TNDR, tumor necrosis factor receptor

TNFR1, tumor necrosis factor receptor 1

TRADD, tumor necrosis factor receptor type 1-associated DEATH domain protein

TRAF, TNF receptor-associated factor

TRAIL, TNF-related apoptosis-inducing ligand

TRAIL-R, TNF-related apoptosis-inducing ligand receptor
