# Supplementary material for: On the intrinsic disorder status of the major players in programmed cell death pathways
Source: F1000Res. 2013 Sep 17;2:190. [Version 1] doi: 10.12688/f1000research.2-190.v1 (PMC3829196; doi:10.12688/f1000research.2-190.v1)
Supplement: Peer review history

# Peer review: Referee response for version 1

**Published:** 2013-10-23

**Authors:** Oxana Galzitskaya

**Affiliations:** 1 Institute of Protein Research, Russian Academy of Sciences, Moscow, Russian Federation

This paper by Uversky *et al.* describes the proteins involved in programmed cell death pathways (apoptosis, autophagy, and necroptosis). Overall it is an interesting paper deserving of publication and contains very exciting illustrative material.

 

The authors have performed a study with a particular accent on the intrinsic disordered status of these proteins. To predict the status of disordered residues, the authors used two programs: PONDR FIT and PONDR® VLXT. It should be noted that there are now programs that work with the same or better accuracy of prediction such as SPINE-D, MFDp, MD, Disopred2, IsUnstruct (version 2.02) and DISOclust (for more details see Table 5 in Lobanov, Sokolovskiy, and Galzitskaya *Journal of Biomolecular Structure & Dynamics* 31(10), 1034-1043, 2013). PONDR®VLXT was the least accurate at disorder prediction according to the AUC of 14 programs that were compared in Lobanov *et al.*

I have read this submission. I believe that I have an appropriate level of expertise to confirm that it is of an acceptable scientific standard.

# Response

**Published:** 2013-10-28

**Authors:** Vladimir Uversky

**Affiliations:** Department of Molecular Medicine, College of Medicine, University of South Florida, USA

First of all, we would like to thank this reviewer for their positive evaluation of our work.

We do agree that the number of available disorder predictors is very large and many of them have accuracies close to that of PONDR FIT. PONDR FIT is a metapredictor representing a consensus of artificial neural network prediction tools, which was developed by combining the outputs of PONDR-VLXT, PONDR-VSL2, PONDR-VL3, FoldIndex, IUPred, and TopIDP. Since PONDR FIT possesses improved prediction accuracy compared to its individual component predictors, and the overall accuracy of this tool is comparable to the accuracies of other meta-predictors, we do not think that the use of additional computational tools is needed for this study. 

We also know that PONDR VLXT is not the most accurate disorder predictor. However, this predictor is extremely sensitive to the local peculiarities of amino acid sequences and therefore, is very useful for finding potential disorder-based interaction sites. This point was specifically discussed in our paper dedicated to the development of the related computational tool, alpha-MoRF-PredII (Cheng Y, Oldfield CJ, Meng J, Romero P, Uversky VN, Dunker AK. Mining alpha-helix-forming molecular recognition features with cross species sequence alignments. *Biochemistry*. 2007; **46**(47): 13468-77. PMID: 17973494).

# Peer review: Referee response for version 1

**Published:** 2013-09-26

**Authors:** Martin Vabulas

**Affiliations:** 1 Institute of Biophysical Chemistry, Goethe Universtiy Frankfurt, Frankfurt, Germany

By means of several computational tools Uversky *et al.* investigates the level of disorder of key members in the cellular network of apoptosis, autophagy, and necroptosis. The study represents an extension of their previous work ( Peng *et al.* 2013) and the two are best read together. I found the study very interesting since it implicates structural disorder as a cell death network building/sustaining principle. Since individual components of the network are analyzed and the results of disorder prediction and connectivity (according to the STRING database) are presented back-to-back the study represents a handy knowledge resource and lays ground for the future experimental validation.

I have read this submission. I believe that I have an appropriate level of expertise to confirm that it is of an acceptable scientific standard.

# Response

**Published:** 2013-10-28

**Authors:** Vladimir Uversky

**Affiliations:** Department of Molecular Medicine, College of Medicine, University of South Florida, USA

We are thankful to Dr. Vabulas for his high evaluation of our work and hope that the readers of F1000Research will find this study useful.
